# Comparing the Effects of Negative and Mixed Emotional Messages on Predicted Occasional Excessive Drinking

**Published:** 2008-01-12

**Authors:** Pilar Carrera, Amparo Caballero, Dolores Muñoz

**Affiliations:** Universidad Autonoma de Madrid (Spain)

**Keywords:** mixed emotions, risk behavior, alcohol use, attitude change

## Abstract

In this work we present two types of emotional message, negative (sadness) versus mixed (joy and sadness), with the aim of studying their differential effect on attitude change and the probability estimated by participants of repeating the behavior of occasional excessive drinking in the near future. The results show that for the group of participants with moderate experience in this behavior the negative message, compared to the mixed one, is associated with higher probability of repeating the risk behavior and a less negative attitude toward it. These results suggest that mixed emotional messages (e.g. joy and sadness messages) could be more effective in campaigns for the prevention of this risk behavior.

In this study we decided to explore a behavior difficult to predict but of enormous social and personal significance to young people: the *occasional excessive consumption of alcohol*. We would like to specify that we are not working here with people with serious alcohol dependency problems; on the contrary, we focus on the young university population which, occasionally, particularly at weekend parties, drinks alcohol in excess. The meaning of *excess* in this research has been defined in a subjective-personal way, as the level that each participant personally considers “excessive”, so that we can take into account individual differences (e.g. weight, habits, type of alcohol, and so on).

In Spain, this type of behavior is frequent and give cause for concern, especially when it is combined with the driving of automobiles. Becoña (2000) points out that in 1988, 80% of school pupils between the ages of 14 and 18 reported that they had drunk alcohol in the previous year; in a recent study with teenagers from Madrid (Spain) aged 14 to 16, 67% reported that they drank alcohol at weekends (Caballero, Carrera, Sánchez, Shih, Muñoz, Toro, Fuente, and Muñoz, 2004). The consumption of alcohol by young people is not a problem exclusive to the Spanish context, but is unfortunately present in many cultures, including Western ones such as the USA (e.g. Johnston, O’Malley and Bachman, 2002) or Asian ones (e.g. Han, Choe, Lee and Lee, 2001).

In general, risk behaviors are evaluated negatively when bipolar measures are used, but this negative attitude does not predict real behavior. Young people report a negative general attitude towards drinking alcohol, but they indulge in it fairly frequently; this could be explained by the fact that pleasant experiences are associated with it in the short term and unpleasant ones in the medium term (Caballero, Carrera, Muñoz and Sánchez, 2007). When unipolar measures were used (positive and negative scales), drinking alcohol showed the highest degree of attitudinal ambivalence in a sample of students (see Conner, Povey, Sparks, James, and Sheperd, 1998). Because attitudinal ambivalence is associated with mixed motives, in health-risk behaviors the correlations between attitudes and intentions (e.g. Urland and Ito, 2007) and between intention and behavior (e.g. McMillan and Conner, 2003) are low or moderate. In other risk behaviors, such as casual sex, Conner and Flesch (2001) found that ambivalence moderated the impact on intentions of affective attitudes, but not of cognitive attitudes (lower ambivalence was associated with a stronger affective attitude-intention relationship).

Adding specific emotions to improve predictions is not new in attitude research (e.g. Carrera, Caballero, Sánchez and Blanco, 2005; Pfister and Böhm, 1992; Sheeran and Orbell, 1999), but to the best of our knowledge, mixed emotions have not been explored as persuasive messages in risk behaviors. In previous research in which we asked participants to recall their personal experience of sex without condom and drinking, we found mixed emotions (joy and sadness) associated with them (Caballero, Carrera, Sánchez, Muñoz, and Blanco, 2003; Caballero et al. 2007; Carrera et al. 2005). Research on mixed emotions, two emotions of opposing valence, shows that they are complex emotional experiences associated with emotional tension (Carrera and Oceja, 2007; Larsen, McGraw, Mellers and Cacioppo, 2004; Oceja and Carrera, 2007; Schimmack, 2001). The negative arousal is the result of bivalent behavioral tendencies of approaching and avoiding (Cacioppo, Gardner and Berntson, 1997) but it may be less negative than the arousal associated with only strong negative appeals, such as fear appeals.

Cacioppo, Gardner and Berntson, (1999) pointed out how mixed emotions could motivate the careful exploration of new settings: the positive feeling fosters exploratory behavior and the negative one maintains attention to avoid threatening events. But if we induce only strong negative emotions, defense (Hovland, Janis, and Kelley, 1953; Leventhal, 1970) and reactance (Brehm and Self, 1989; Fuegen and Brehm, 2004) are the most frequent responses (e.g. to fear messages). One strategy for reducing defense and reactance is to present fear appeals accompanied by solutions which are considered easy to carry out (Petty, DeSteno and Rucker, 2001). If we use only strong fear appeals we shall find the opposite effect to the desired one, so that the practice of the risk behavior will increase and will even be evaluated more positively (Simon, Greenberg and Brehm, 1995). In general relevant groups (high frequency in risk behavior) are often the least persuade by threatening health appeals (e.g. Morris and Swann, 1996). Here we shall explore another strategy for reducing intention to perform the risk behavior: adding a positive emotional message to a negative emotional one. We expect the positive emotion in the mixed appeal to reduce the negative arousal provoked by the negative information and increase interest in changing the risk behavior, thus reducing the intention to repeat the risk behavior in the near future. This relationship will be stronger in the group with high levels of experience of drinking alcohol in excess because this sample will probably feel higher reactance to strong negative emotional appeals.

In order to explore the role of these differences in the intention to repeat occasional excessive drinking we manipulate mixed versus negative emotional messages. We chose joy and sadness (mixed emotion) versus sadness as emotional messages because they were the emotions most commonly associated with drinking behavior in our previous research (see Carrera et al. 2005). We expect that, compared to the negative emotional message, the mixed emotional message does not change the previous negative attitude, but encourages participants to reconsider repeating the conduct in the immediate future. This effect is expected in people with a moderate-high level of frequency in the risk behavior, a group in which the dissonance will be greater and where the rejection of negative emotional messages is likely to be higher. In the group of people that do not usually drink alcohol in excess we do not expect any effect from the two types of emotional message (negative versus mixed), given that in these people it is probable that “not drinking” is an established habit, coherent with their attitudes, so that it is possible that they do not attend to the message. Personal experience in risk behavior will be explored as moderator in the relationship between the two emotional messages (mixed versus negative) and the intention to drink in the following weeks.

## Method

### Participants

Ninety-three students from the Psychology Department at the Universidad Autonoma de Madrid took part in this study, with an average age of 19.6 years; 17 were males and 76 were females. Participation was voluntary and anonymous. Participants were randomly assigned to the two experimental conditions, 45 participants (10 male and 35 female) to the negative message and 49 participants (7 male and 41 female) to the mixed message.

### Procedure

All participants voluntarily completed the survey which was made up of two parts, before and after the emotional message. The survey was carried out in individual, isolated booths so that participants could concentrate on the task in hand, particularly in the second part where the intention was to induce emotions via the verbal (written) messages. Once the first section of questions had been completed, a research assistant gave them the message and the second part of the survey. In the first part of the survey, the following was measured: participants’ general attitude towards *drinking excessive alcohol*, without specifying a quantity, so that each individual could apply their own criteria as to what they considered excessive. We chose a subjective definition of *excess* because in previous studies we found substantial individual differences in the quantity of alcohol young people considered excessive (see Caballero et al. 2004). For the measurement of attitude we used two unipolar 7-point scales, measuring positive evaluation and negative evaluation. Together with the attitude measurements, the question was also posed about the *frequency* with which participants drank alcohol in excess on a 7-point scale (ranging from *never* to *very frequently*). This measurement is used later as a moderator variable in the analysis. In the second part of the survey, after carefully reading the emotional message, participants were asked to respond to five strictly unipolar scales about the emotions felt after reading the message (*joy, calm, tension, sadness, anger and fear*), first answering yes/no as to whether they had felt that emotion, and only in the case of an affirmative answer with what intensity they had felt it on a 7-point scale (from *not at all* to *very intensely*). Participants were assigned randomly to the two experimental conditions (i.e. negative message or mixed message). Following the questions about the emotional experience, they once again rated the behavior of drinking excessive alcohol, this time with an open-closed question, so that they were first asked to write whatever came to mind at that moment about the behavior and, after writing it down, to rate their ideas on two unipolar 7-point scales (to what extent were the written ideas positive and/or negative). This change of format in the measurement of attitude was aimed at dissuading participants from automatically reproducing the same values that they had marked on the scales before reading the message. After filling out the scales on attitude they were asked about the *probability of performing the behavior of drinking excessive alcohol* in the following weeks, indicating their response on a 7-point scale (from 1 *none* to 7 *highly probable*). We decided to induce sadness with the negative message and joy and sadness with the mixed one. We chose sadness rather than fear or anger, as previous studies on this behavior with a similar group (young people aged between 18 and 20) showed that it was the emotion most frequently associated with the memory of having performed this risk behavior in the past (Carrera, Caballero, Sánchez and Blanco, 2005). Negative and mixed messages are included in Appendix 1.

## Results

First, we evaluated the effectiveness of our messages for inducing emotions (sadness and mixed). In the negative message 43 (9 men and 34 women) of the 45 participants reported having felt the emotion of sadness but not of joy; in the mixed message 36 (4 males and 32 females) of the 49 participants reported having felt joy and sadness on reading the message. In the analysis we use only the samples that fit the manipulation of the independent variable (43 participants in the negative message and 36 in the mixed one). Contrary to what we had expected, there was no significant difference in the intensity of sadness between those who only experienced the negative emotion, negative condition (*M* =5.26, *SD* = 1.5) and those who experienced it together with joy, mixed condition (*M* = 5.34, *SD* =1.45), *t* (75) = 0.023, *p* >0.05, *d* = 0.05. Neither were there differences in the category of tension between the negative condition (*M* = 3.7, *SD* = 1.7) and the mixed one (*M* = 4.01, *SD* = 1.5), *t*(52) = 0.72, *p>* 0.05, *d* = 0.19. The categories of *anger, fear, calm* and *tension* had been included to avoid suspicion about the experimental hypotheses and to control acquiescence bias. These categories were experienced by 61 participants in the case of anger, by 34 in that of fear, by 14 in that of calm and by 60 in the case of tension. Only 4 participants said they felt calm and at the same time tension, which is considered as confirmation that acquiescence bias did not apply.

Next, we calculated the attitudinal measurements before and after the messages in both groups. The two attitude scales (negative and positive re-coded) pre-message presented a good index of reliability (Cronbach’s *alpha* = 0.70); in the case of the post-message attitudes (negative and positive re-coded), these also showed appropriate levels of reliability (Cronbach’s *alpha* = 0.92). We calculated the general attitude as the average of the two scales, re-coding the positive one. In the sample that received the negative message and reported having felt sadness, we obtained a significant change of attitude, the attitude after the message (*M* = 5.72, *SD* = 1.6) being less negative than before (*M* = 6.32, *SD* = 0.73), *t* (41) = 2.43, *p*<0.01, *d* = 0.48 (a medium effect). This result confirmed the hypothesis that when faced with strong negative messages those with experience of the behavior reject them and opt for the least costly strategy, changing the evaluation of the behavior. In the sample that received the mixed message and reported having felt joy and sadness there was no significant change between the general attitude before (*M* = 6.44, *SD* = 0.75) and after the message (*M* = 6.08, *SD* = 1.5); *t* (34) = 1.8, *p*>0.05, *d* = 0.30. These results show that whereas there was a change of attitude in participants when faced with the negative message, this was not the case for the mixed message. It seems that the positive emotion in the mixed message has acted as an ‘antidote’ to the dissonance generated by the priming of the counter-attitudinal behavior and the negative emotion also induced in the mixed message. This difference cannot be explained by the amount of previous experience in the sub-samples, since in either case it was moderate (in sample with negative message *M* = 2.9, *SD* = 1.4, and in sample with mixed message *M* = 2.8, *SD* = 1.5), and similar in the two cases (*t* (77) = 0.05 *p*>0.05, *d* = 0.06).

**Figure 1:**
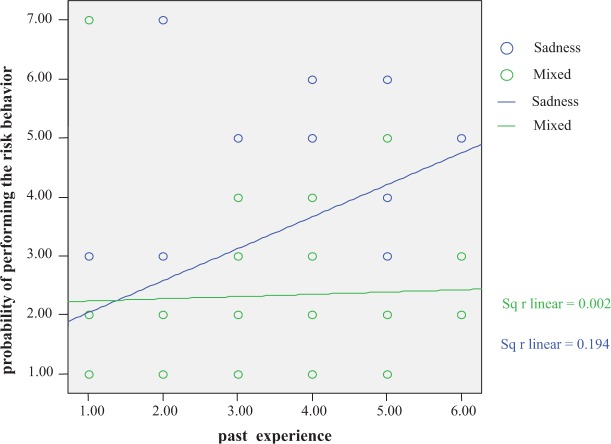
The probability of performing the behavior of excessive drinking in the coming weeks as a result of past experience in the behavior and the type of emotional message received.

As well as the effect of the negative and mixed emotional messages on attitude, we also wanted to know: 1) their influence on the probability of performing the risk behavior in the immediate future as estimated by the participants, and 2) the moderating role of experience in the behavior on that relationship. We standardized all the variables to carry out a hierarchical regression analysis on the probability of performing the excessive drinking behavior in the following weeks, with the type of emotional message (negative vs. mixed) and experience in carrying out this risk behavior as predicting variables. The main effects were obtained in the first step of the regression and the interaction in the second step, introducing the product between the two independent variables (see Cohen and Cohen, 1983). The results of this analysis revealed a main effect of frequency with which the participants carried out the risk behavior, *β* = 0.28, *t* (75) = 2.58, *p<* 0.01, but not of the type of emotional message, *β* = −0.19, *t* (75) = −1.83, *p* = 0.07. However, the interaction between the type of emotional message and participants’ frequency of performing the risk behavior was significant, *β* = −0.20, *t* (74) = −1.92, *p* <0.05. These results indicate that although the emotional messages did not have a differential effect in people with little experience in the risk behavior, they did have an effect on those with greater experience, so that the participants with more experience who received the negative message estimated that they would be more likely to participate in the risk behavior in the near future, as can be seen in Figure 1.

Participants with experience in the risk behavior, on seeing the negative message, indicated that the probability of their continuing to perform the behavior was higher than those who received the mixed message. Moreover, the participants with greater experience who received the mixed message indicated a low probability of repeating the risk behavior, as low as that of participants with little experience of carrying out the behavior.

After checking that the interaction between frequency in the behavior and the type of emotional message was significant, we carried out a regression analysis of the frequency in the risk behavior on the probability of carrying it out in the near future, both for the negative message and for the mixed message; in this way we could find out the effect of past experience in the behavior for each one of the messages. The results indicate that in the group that received the negative message, there was a clear effect of experience in the behavior: the greater the experience, the higher the probability of carrying out the behavior *β* = 0.44, *t* (41) =3.14, *p*<0.003. In the mixed message, experience had no effect *β* = 0.04, *t* (34) = 0.23, *p* = 0.81. We dichotomized the sample by the median, (*Md* = 3), high (>=3) and low (<=2) in frequency of carrying out the risk behavior, finding that those who reported a high level of experience in drinking excessive alcohol and who received the negative message, feeling sadness, reported a greater probability of carrying out the risk behavior (*M* = 3.6, *SD* = 1.5) than those who received a mixed message and felt joy and sadness (*M* = 2.45, *SD* = 1.1); for this group (high experience) the regression analysis of type of message on the probability of carrying out the behavior was significant, *β* = −0.30, *t* (47) = −2.2, *p* <0.03. In participants with low experience of drinking excessive alcohol the regression analysis did not reveal significant differences in the probability of excessive drinking after the negative message (*M* = 2.3, *SD* = 1.7) or the mixed message (*M* = 2.12, *SD* = 1.9), *β* = −0.05, *t* (41) = −0.35, *p* = 0.72.

## Discussion

In this research we were interested in evaluating whether adding a positive emotion to negative emotional messages about risk behavior (i.e. mixed appeal) achieved a reduction in the usual rejection produced by strong negative emotional appeals. This effect would be produced by a larger reduction of the negative activation in the mixed message compared to the negative one. We expected that in the mixed condition the classic boomerang effect would be collapsed. This effect would be higher in the sample that frequently repeated the risk behavior because the dissonance in them would also be stronger. This would mean that participants with high frequency in occasional excessive drinking of alcohol who participated in the mixed condition would maintain their previous negative attitude towards the risk behavior (not moving toward the positive pole), but would reduce their intention to carry it out in the near future. These results were not expected in the sample without experience and in the group in which only sadness was induced.

Our results clearly showed that the two types of emotional messages, negative versus mixed, had a differential effect: a) on the change of attitude (pre-post message) and, b) on the estimated probability of repeating the risk behavior in the immediate future. As we expected, the influence of the messages on the probability of carrying out the behavior in the near future was moderated by participants’ experience in the risk behavior, but we did not obtain the same moderating effect on the change of attitude. The messages produced differential results only in people who had carried out the behavior in the past with moderate frequency.

For the group with high-moderate experience in the risk behavior, the results showed that the negative message provoked a higher estimation of the probability of repeating the risk behavior, which they evaluated more positively than before reading the emotional message. This data is coherent with the hypothesis of reactance and emotional control (Hovland et. al. 1953; Leventhal, 1970). However, the mixed message did not cause the same reaction; on the contrary, the estimates of these participants on their probability of drinking excessive alcohol in the following weeks was as low as the estimates of those who did not usually drink alcohol. These results also appear to be substantiated by the data obtained about the average attitude after the message. In the group which received the negative message it was moderated, becoming less negative than before the message, whereas it remained equally negative in the group that received the mixed message.

In spite of the differences found in attitude and the probability of carrying out the behavior, we did not obtain the expected result in the proposed mediator: the lower negative activation in the mixed message. Similar levels of intensity of sadness and tension were obtained in the two messages. It may be that there was a ceiling effect in the measurement, or that the categories selected were not appropriate.

Of course, a limitation of our work is the lack of a control group with which to evaluate attitude and probability of repeating the behavior after reporting attitude and past behavior, but without an emotional message. In numerous risk behaviors the attitudes are appropriate (i.e. negative), and what generates the problem is that neither intentions nor behavior are coherent with them, so that research efforts should focus on how to change these intentions and behavior.

## Appendix 1

Negative Message (sadness): “Juan was drinking with his friends. He drank a lot, then he got into his car to take his girlfriend home, thinking he was OK to drive. On a bend he wasn’t able to react in time and crashed into a vehicle coming in the opposite direction. All of them died instantly.”

Mixed Message (joy and sadness): “Juan managed to get the grant he so desired, at last he would be able to study what he had always dreamt about and, moreover, could share a flat with his girlfriend who had already moved in and was there waiting for him. To celebrate, he arranged to meet his friends and drank a lot. Then he got into his car and on a bend in the road he lost control. He died instantly.”
